# A new method to transfect the hypoblast of the chick embryo reveals conservation of the regulation of an Otx2 enhancer between mouse and chick extraembryonic endoderm

**DOI:** 10.1186/1471-213X-7-25

**Published:** 2007-04-02

**Authors:** Amanda Albazerchi, Olivier Cinquin, Claudio D Stern

**Affiliations:** 1 Department of Anatomy and Developmental Biology, University College London, Gower Street, London WC1E 6BT. UK; 2 Laboratory of Genetics, 425-G Henry Mall, University of Wisconsin, Madison, WI 53706, USA; 3 Deparment of Biochemistry, University of Wisconsin, Madison, WI 53706, USA

## Abstract

**Background:**

The mouse anterior visceral endoderm (AVE) and the chick hypoblast are thought to have homologous roles in the early stages of neural induction and primitive streak formation. In mouse, many regulatory elements directing gene expression to the AVE have been identified. However, there is no technique to introduce DNA into the chick hypoblast that would enable a comparison of their activity and this has hampered a direct comparison of the regulation of gene expression in the mouse and chick extraembryonic endoderm.

**Results:**

Here we describe a new method to introduce DNA into the chick hypoblast, using lipofectamine-mediated transfection. We show that the hypoblast can be easily transfected and that it starts to express a luciferase reporter within 2 hours of transfection. The validity of technique is tested by following the movement and fate of hypoblast cells, which reveals their translocation to the anterior germinal crescent. We then introduce a vector containing GFP driven by the mouse VEcis-Otx2 enhancer (which directs gene expression to the mouse AVE) and we detect activity in the hypoblast.

**Conclusion:**

The new technique for delivering expression constructs to the chick hypoblast will enable studies on gene activity and regulation to be performed in this tissue, which has proved difficult to transfect by electroporation. Our findings also reveal that regulatory elements that direct gene expression to the mouse AVE are active in chick hypoblast, supporting the idea that these two tissues have homologous functions.

## Background

Comparing the initial stages of neural induction and gastrulation in avian and murine embryos highlights evolutionary conservation of mechanisms responsible for directing the early events of embryonic development. During these stages, the appearance of the two organisms is quite distinct: the chick embryo forms from a flat disc whilst the mouse develops from a cylindrical epiblast. Both species possess extraembryonic endodermal tissues whose fate is to contribute to structures such as the yolk sac stalk but not to the embryo proper [1,2]. In the chick, these tissues include the hypoblast and the endoblast [3,4], whereas their mouse counterparts are most likely the Anterior Visceral Endoderm (AVE) and the rest of the Visceral Endoderm (VE), respectively. The avian hypoblast is molecularly and functionally homologous to the mouse anterior visceral endoderm (AVE) in that both act to restrict the site of primitive streak formation [4,5] by inhibiting Nodal signalling.

To analyse the function of the AVE in mouse, transgenic techniques can be employed to create a gain-of-function, producing embryos expressing a gene of interest under the control of an AVE-specific enhancer. Loss-of-function analysis is achieved by generating null mutations in the AVE through the injection of wild-type ES cells into mutant blastocysts [6]. Recently the chick has become amenable to gain- and loss-of-function experiments through electroporation of expression constructs and morpholinos or RNAi molecules targeted against specific mRNAs [7-9]. However, loosely associated cell populations such as the hypoblast do not respond well to this technique leaving it without means of genetic manipulation. Consequently, the role of genes expressed in the hypoblast, and the conservation of their regulation with those in the mouse AVE, have been impossible to test directly.

Here we describe a method based on lipofectamine-mediated delivery of DNA into the hypoblast. The hypoblast responds efficiently to this treatment and a 0.5–1 hour exposure to the transfection medium results in strong expression of reporter constructs. This method is used to analyse the activation of a mouse Otx2 enhancer previously shown to drive expression exclusively in the mouse AVE prior to primitive streak formation and is later active in the anterior definitive endoderm (ADE) and anterior mesendoderm [10,11]. The enhancer shows a similar pattern of activation in the chick suggesting a conserved mechanism for the regulation of Otx2 expression in murine AVE and avian hypoblast.

## Results

### The hypoblast can be transfected within 1 hour and starts expressing within 2 hours

Previous experiments using lipofectamine to introduce DNA into embryonic tissue have involved the injection of a lipofectamine-DNA mix directly into the region of interest [12,13]. However, at pre-primitive streak stages, the hypoblast and embryo both disintegrate quite rapidly after this treatment. The problem with conventional cell culture lipofection protocols is that they require more than 3 hours' exposure to the reagent to obtain a high transfection rate. The hypoblast traverses the embryo in a matter of hours and a three-hour period might affect its function and integrity.

To optimise transfection efficiency whilst minimising hypoblast degradation and length of transfection time, two factors were altered: the Lipofectamine-complex:DMEM ratio, and transfection time. This is presented in Table 1. Since the hypoblast inhibits primitive streak formation [4] and directs the movements of the prospective fore/midbrain [3,14,15], a 'successful embryo' was classified as one in which the embryo developed with a normal axis extension and orientation. As shown in Table 1, the optimal outcome was achieved using conditions E and F but because of the shorter time of transfection, E was prefered. All subsequent experiments were performed under these conditions.

**Table 1:** Comparison of transfection efficiencies

***Set***	***% Lipo-complex***	***Transfection time***	***n***	***(n) % efficiency***	***Level of expression***	***(n) % embryo success***
A	33%	30 min	8	(4/8) 50%	low	(4/4) 100%
B		60 min	6	(5/6) 83%	low	(4/5) 80%
C		90 min	9	(9/9) 100%	medium	(9/9) 100%
D	66%	30 min	8	(4/8) 50%	medium	(3/4) 75%
E		60 min	7	(7/7) 100%	high	(5/7) 71%
F		90 min	8	(8/8) 100%	high	(6/8) 75%
G	100%	30 min	6	(5/6) 83%	medium	(2/5) 40%
H		60 min	7	(7/7) 100%	high	(3/7) 43%
I		90 min	8	(8/8) 100%	high	(1/8) 13%

Different combinations of transfection period and Lipofectamine-complex were tested. The efficiency of transfection is based on the number of embryos in which fluorescence could be detected following overnight culture. The 'level of expression' is an estimate of the proportion of cells expressing the reporter. Finally, the normality of the embryos ('embryo success') with fluorescent hypoblasts was judged as embryos that developed with a normal axis extension and orientation and in which the hypoblast developed in a characteristic fashion.

**Figure 1:**
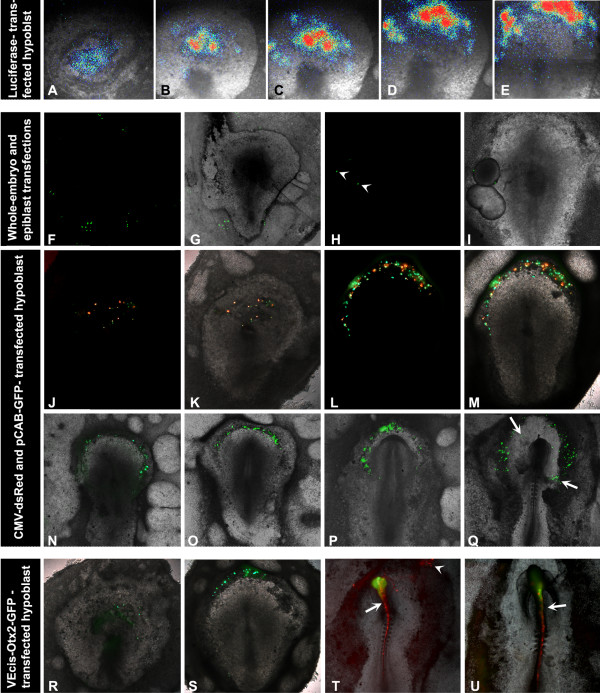
Transfection of the hypoblast. **A-E: **pCAB-Luc monitored by luciferase detection at time points following hypoblast tranfection: 2 hours (A), 5 hours (B), 7 hours (C), 10 hours (D), 14 hours (E). Coloured pixels represent light intensity with blue being least intense and red most intense. **F-M: **GFP expression in whole embryo- (F,G) and epiblast-transfected (H,I) tissues (fluorescence images, F, H and overlaid with brightfield images, G, I). Arrowheads indicate cells expressing GFP in H. **J-M: **dsRed and GFP expression in the hypoblast 8 hours (J,K) and 14 hours (L,M) after transfection. An overlay of fluorescence and brightfield images are shown in K and M. **N-Q: **longer culture periods reveal the transfected hypoblast (shown here with GFP only) in stage HH5 (N), HH7 (O), HH8- (P) and HH9 (Q) embryos. Arrows indicate labelled cells in the yolk sac. **R-S: **VEcis-Otx2 driving GFP is detected in transfected hypoblasts after 7–8 hours (R) and is seen in the crescent-shaped hypoblast at stage 4+/5 (S). **T-U: **Co-electroporation of VEcis-Otx2-GFP and CMV-dsRed shows GFP localised in the embryo in the rostral to the hindbrain (T) and also occasionally in the hindbrain (U) (arrows). dsRed is expressed in the entire electroporated region and is seen more caudally in the embryo and in the area opaca (arrowhead).

To estimate the timing of translation initiation from the point of transfection, a luciferase reporter contruct was created using the ubiquitous chicken β-actin promoter (pCAB-Luc). pCAB-Luc was transfected into the hypoblast. Localised luciferase expression was detected after 1–1.5 hours of filming (1.5–2 hours after placing the embryo in culture: Fig. 1A). From this point on, the signal increased in strength and followed the anteriorly-directed movement of the hypoblast (Fig. 1A–E, different embryo: Additional file 1).

### Lipofectamine selectively transfects hypoblast cells

Whole embryos, epiblast alone or hypoblast alone, all taken at stages XII-XIII, were tested for their susceptibility to lipofectamine transfection. The optimal conditions, as ascertained above, were used to transfect tissues with a GFP reporter under the β-actin promoter (pCAB-GFP). Following transfection, epiblasts and hypoblasts were grafted onto host embryos and all embryos were incubated overnight. In embryos treated as a whole, expression of GFP was detected occasionally in the yolky cells of the extra-embryonic area opaca (3/10; Fig. 1F, G). Grafts of transfected epiblasts exhibited expression in one instance out of 10 embryos, and then in only two cells (Fig. 1H, I). In contrast, hypoblasts were transfected successfully (3/4). It is therefore likely that all expression seen in embryos following a graft of a transfected hypoblast arises from the initial transfection of this tissue rather than from transfer of the lipofectamine-DNA complex into the epiblast.

### Transfection of reporter constructs does not affect hypoblast movements or fate

The reporter-expressing hypoblast develops in a characteristic fashion, spreading across the entire area pellucida at pre-primitive streak stages and then being displaced in a posterior-to-anterior direction to become located in a crescent shaped region (the germinal crescent) beneath the prospective rostral neural plate at full primitive streak stages (Fig. 1A–E, J–M), and can still be found in this position at headfold and somite stages (Fig. 1N–P). Embryos with transfected hypoblasts developed to a maximum of stage HH9 (approximately 38 hours in culture; Fig. 1Q). At this stage labelled cells surround the whole head region of the embryo as they are incorporated into the yolk sac stalk [1]. This is similar to the fate of the visceral endoderm in mouse [2].

### Mouse Otx2 AVE enhancer drives expression in the hypoblast

To test whether regulatory mechanisms driving gene expression in the mouse AVE are conserved in the chick hypoblast, we used an Otx2 enhancer (VEcis-Otx2) shown in the mouse to be active exclusively AVE at pre-primitive streak stages and later in the ADE and anterior mesendoderm [10,11]. A VEcis-Otx2-GFP construct was transfected into the hypoblast and GFP expression was detected from the earliest time point (7 hours post-transfection: Fig. 1R) and persisted during all stages analysed (until stage HH6-7). This is beyond the stage at which *Otx2 *is normally expressed in the hypoblast [16] (Fig. 1S). The persistence of fluorescence is most likely due to the long stability of this protein. However, to rule out the possiblity that the enhancer promiscuously drives expression throughout the embryo, it was introduced into the embryonic epiblast and the area opaca of stage HH3 embryos by electroporation. Expression of GFP was restricted to the head region of the embryo (10/10; Fig. 1T, U) and with a caudal boundary of the midbrain-hindbrain border (9/10), reminiscent of the normal pattern of expression of *Otx2*. No expression was detected in the area opaca or in the embryo caudal to the hindbrain. In one case hindbrain expression was detected (Fig. 1U), which again could be the result of persistent GFP in cells that originally expressed *Otx2*.

## Discussion

The method described here makes the chick extraembryonic endoderm amenable to genetic manipulation and to the study of the activity of enhancers directing expression to this tissue. Several genes are known to be expressed in the hypoblast including Cerberus (shown in *Xenopus *to be a multi-functional Wnt, Nodal and BMP antagonist [17]), Wnt angagonists Dkk1 and Crescent, FGF8 and Otx2 [3,4,18,19]. FGF and retinoic acid signalling as well as Wnt- and BMP-inhibition, have also been implicated in the ability of the hypoblast to induce expression of *Otx2*, *Sox3*, *Cyp26A1 *and *ERNI *in the epiblast [20] but it has not been possible as yet to demonstrate their requirement by loss-of-function experiments. The technique described here will enable gain- and loss-of-function experiments to be performed to assess the role of the hypoblast in crucial events prior to and during gastrulation. It has so far been possible to graft beads coated in various factors or cells secreting proteins to interfere with signalling pathways. However, the advantage of the method described here is that it allows tissue-specific targeting thereby avoiding difficulties in interpretation of an epiblast- versus hypoblast-mediated effect. Furthermore, it allows transcription factors to be overexpressed, or knocked down with a morpholino or by RNA interference. Using a luciferase reporter, protein translation was shown to be initiated within 2 hours of transfection, which would allow the hypoblast to express the gene before the primitive streak starts to form.

The characteristic movement of the hypoblast, from a position underlying the entire embryonic epiblast to becoming restricted to the germinal crescent, is recapitulated here with transfected hypoblasts, which undergo normal movements. Following the longest periods of culture, some labelled cells of the hypoblast are found in the prospective yolk sac stalk, consistent with the results of Rosenquist [1] and comparable to the fate of mouse visceral endoderm [2].

The transfection technique was also used to assess the activity of an element that drives *Otx2 *expression in the anterior visceral endoderm in mouse [10]. This element was found to be active in the chick hypoblast and to drive expression of GFP indicating a common mechanism for Otx2 activation between the two species. Using this technique, further comparative analysis of regulatory elements could be performed.

## Conclusion

The method proposed here enables introduction of DNA into the avian extra-embryonic endoderm. The chick embryo culture system is ideal for imaging real-time expression of fluorescent proteins. The procedure itself does not adversely affect the embryo and allows genetic manipulation of the hypoblast. Furthermore, the conservation of regulatory elements can now be tested. We show that a mouse Otx2 enhancer, active in the AVE, can also drive expression in the hypoblast, revealing a conservation of elements driving gene expression in these tissues in the two species.

## Methods

### Constructs

The ability of both the CMV enhancer and promoter and the chick B-actin promoter with a CMV enhancer (pCAB) to drive expression were tested using CMV-dsRed reporter construct (dsRed Express N1: Clontech) and pCAB-GFP (gift of A. Lumsden; see [9]). Fluorescence was not observable within 5–6 hours. This is potentially due to the nature of post-translational modifications of fluorescent proteins, which require 2–4 hours to fold before fluorescence can be detected [21]. To establish that translation of protein could commence in a shorter time frame following transfection, a pCAB-Luc construct was used to drive expression of Firefly luciferase, which is active much sooner after translation.

The VEcis-Otx2 construct [11] drives expression of GFP in the mouse AVE, anterior definitive endoderm and anterior mesendoderm [10] and was tested here for its ability to drive expression in the hypoblast and other regions of the chick embryo.

### Dissections

Eggs (Henry Stewart, UK) were incubated until stage XII-XIII (Roman numerals for pre-primitive streak stages [22]). They were transferred to modified New culture [23].

Whole embryos were transfected by removal from the vitelline membrane and placed in a 250 μl drop of transfection medium (see below for full details) Epiblasts and hypoblasts were dissected away from the embryo and transfected in the same way. Subsequently, transfected epiblasts were grafted to an ectopic site at the boundary between the area opaca and area pellucida of a host embryo; hypoblasts were returned to the embryo from which they had been dissected.

### Transfections

Transfection medium was prepared by varying the proportions of the cell transfection protocol in which 1 μg DNA, 6 μl Plus reagent (Invitrogen), 4 μl lipofectamine (Invitrogen) and 200 μl Optimem (GIBCO) were added to 800 μl DMEM (GIBCO) covering a 35 mm plate. In this study, the ratio of Optimem-Plus-Lipofectamine compound (refered to subsequently as Lipofectamine complex) to DMEM was varied, as was the incubation time. 2 μg DNA was found to be slightly more efficient in all combinations and so was used throughout.

Tissues were placed in the transfection medium and transferred to a tissue culture incubator (37°C, 5% CO2, 100% humidity). After incubation for the required amount of time, they were replaced and the whole culture incubated at 38°C. When embryos were to be imaged for luciferase expression, the thin albumin used for the New culture was supplemented with 50 nM luciferin (Sigma).

### Electroporation

Eggs were incubated until stage HH3 (Arabic numerals for primitive streak and older stages [24]). They were transferred to modifed New culture and the embryos electroporated with VEcis-Otx2 at a final concentration of 1 μg/μl with the aim of transfecting a large region. This was co-electroporated with CMV-dsRed (used at a final concentration of 1 μg/μl) to visualise the electroporated region. To ensure that all regions of the embryo were analysed, embryos were electroporated in the area opaca, posterior epiblast, anterior epiblast and along the midline.

### Analysis

Embryos were analysed under a dissection microscope with fluorescence for expression of dsRed and GFP and with an Imaging Photon detector (IPD); Photek, UK (Sofware and system design provided by Science Wares) for luciferase detection.

## Authors' contributions

All work was performed by AA except the cloning of pCAB-luc, which was carried out by OC. AA and OC carried out luciferase imaging experiments. Experiments were performed in the laboratory of CDS who also participated in the preparation of the manuscript. All authors have read and approved the final version of the manuscript.

## Supplementary Material

Additional file 1 Embryo cultured with hypoblast transfected with pCAB-Luc. This movie sequence covers 12 hours of development immediately following transfection, and shows activity of the luciferase reporter and movements of the hypoblast. Click here for file
